# Out of this world: can surgery learn from NASA’s approach to leadership and safety culture?

**DOI:** 10.1308/rcsann.2025.0079

**Published:** 2025-11-11

**Authors:** O Al-Gholmy, M Davidson, ES Brennan, R Kerstein, PA Brennan

**Affiliations:** ^1^ University Hospitals Southampton, UK; ^2^ BALPA House, UK; ^3^ Bristol Medical School, UK; ^4^ Buckingham Healthcare NHS Trust, UK; ^5^ Portsmouth Hospitals University Trust, UK

**Keywords:** Safety culture, Just culture, Error, Surgery, Human factors, NASA

## Abstract

The US National Aeronautics and Space Administration (NASA) will be familiar to most across the world. Leading highly dangerous and innovative space travel, NASA has gone from a blame culture in the 1960s to an environment that keeps safety at the forefront and a top priority. NASA culture aims to ensure that staff work safely through balancing challenges and risks, feel comfortable communicating safety issues and learn from both successes and error. Surgery, and healthcare in general, still has a long way to go to embed a safety culture that values staff and looks at why incidents and errors have happened, and what can be learnt from them, instead of who was to blame. NASA’s safety journey is a powerful study in learning from failure, evolving culture and leading with humility. From the Apollo 1, Challenger and Columbia disasters, NASA built a more transparent, accountable and resilient safety system and one that continues to evolve with the frontiers of space exploration. In many ways, surgeons can learn a lot from NASA to improve both patient safety and culture.

## Introduction

While surgeons are responsible for their own behaviour and actions, and most do not go to work with the intent of causing error or patient harm, yet healthcare has long been focused on individual accountability. In contrast, most safety-critical industries, otherwise known as high-reliability organisations (HROs), that manage risk in complex, high-stakes environments adopt a Just Culture. This leadership and safety culture approach focuses on why an error has happened and how it can be minimised in future, rather than apportioning blame. As an HRO itself, the UK NHS and other healthcare systems around the world are still a long way behind others in embedding a Just Culture. This does not absolve individuals from actions with the intent of causing harm or compromising patient safety, but fortunately such incidents are rare. Recent examples in the UK include Dr Harold Shipman and the breast surgeon, Ian Paterson.^1^

In contrast, as many as 1 in 20 hospital admissions has some form of unintended error, with 1 in 400 admissions having a serious medical or surgical error.^2^ The operating theatre is one of the commonest areas of the hospital in which error occurs.^2^ Most of these errors are potentially preventable by understanding and applying human factors and having robust systems in place.^3^

Sadly, in healthcare, this culture and way of thinking is still at a relatively premature stage. The Francis Report and Ockenden Review and other similar inquires have identified serious issues with culture in the UK and have initiated changes to practice, although this is not widespread across the NHS.^4,5^ However, many individuals still feel personally blamed for adverse events. This culture restricts transparency and limits institutional learning. By looking at the safety and culture of other HROs, we can learn lessons and appreciate how others have made their own journeys to improvement.

## NASA’s history in challenging the culture of blame

**Figure 1:**
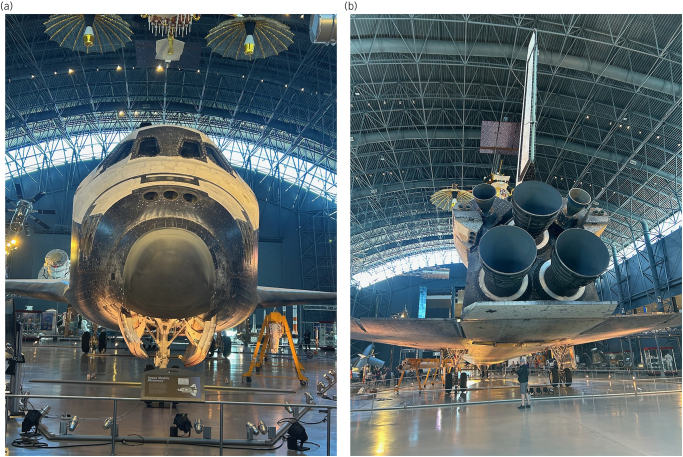
NASA space shuttle Discovery, now housed in the National Air and Space Museum, Fairfax, Virginia USA. She flew 39 Space Missions before retirement. (a) Space Shuttle front end. (b) Space shuttle rear end.

The US National Aeronautics and Space Administration (NASA) is a very successful organisation, known worldwide for its high-stakes and safety-critical operations. NASA put the first man on the Moon and was responsible for the Space Shuttle programme, which was retired in 2011 (Figure 1). From an early stage, NASA recognised that failures tend to be the result of system-level vulnerabilities rather than a single factor. From the 1970 Apollo 13 disaster (with no loss of life) to the tragic 1986 Challenger Space Shuttle explosion 73 seconds after launch (with the loss of seven crew members), NASA’s response to catastrophe has been to investigate thoroughly and learn from system failures rather than apportion blame on individuals. In many ways, surgery can learn from the NASA approach.

In the early 1960s, NASA had a blame culture when incidents happened. However, from as early as 1967 following the Apollo 1 disaster where, during a launchpad test, a fatal fire broke out in the command module killing all three astronauts, NASA changed its approach to investigating incidents and abolished the blame culture, replacing it with a system of identifying systemic weaknesses. They highlighted failures in safety oversight, leadership structure and poor communication.^6^ This incident became a key moment in the development of systems thinking in other HROs. This approach is supported by many. The well-known James Reason (familiar to many for his concept of the Swiss Cheese Model of error) wrote ‘we cannot change the human condition, but we can change the conditions under which humans work’.^7^

NASA now has an enviable leadership and Safety Culture Model that is based on five criteria including active reporting, Just Culture, flexibility, learning and engagement. Further information is available on its safety culture webpages.^8^

NASA has been inspired by this model in all aspects of its missions. Before each launch, flight directors and engineers review mission plans to find any potential problems. These can include ambiguous procedures, single-point failures or equipment vulnerabilities. Any pitfalls are addressed well in advance, hence minimising the risk of error. This method is all part of NASA’s culture of anticipating failure instead of reacting to it. At a safety day in April 2024, held at NASA’s Armstrong Flight Research Centre in Edwards Air-Force Base, California, the NASA Safety Centre Deputy Director (Bob Conway) said ‘Culture is the way work gets done. Everyone is a leader. No accident occurs in the moment. It is the result of a series of events that may be years in the making’.^9^

In surgery, pitfalls can be similar to those of NASA. Examples potentially could include incomplete consent forms, poor handovers, disengagement with briefings and checklists, steep authority gradients and poor communication between other team members.^3,10,11^ While some of these issues in isolation may not have unintended consequences themselves, when grouped together, they can potentially lead to patient harm.

## Learning from failure: a NASA model

The 1986 Challenger Space Shuttle disaster led to a detailed and thorough investigation that was published as the Rogers Commission Report.^12^ It stated that systemic pressures had led to poor decision making. There were clear concerns about O-ring design and erosion on the booster rockets, but these issues were put aside due to launch schedule pressures. Sadly, an O-ring failure led to a leak of hot gases, which in turn led to the fatal explosion. NASA’s review of the disaster did not lead to reprimanding of individuals, but instead, analysed the chain of decisions that led to this issue being overlooked before launch.^13^ Healthcare should be inspired by NASAs postflight analysis reviews, which follow a clear structure, and guidance on how to involve quieter crew members who may be slow to speak up in subsequent discussion and learning meetings.^13^

In surgical practice, and in the context of a Just Culture, an ideal mortality and morbidity meeting (M&M) or serious incident review might look similar; the process should allow staff to feel able and safe to report, without fear of retribution, adverse events and near misses (as happens in NASA). This would be followed by careful case selection (for M&M), excluding those that are attributable solely to individual error. Cases should be discussed openly with involved members and should be reviewed using a selected framework with defined outcomes and learning points. Perhaps most importantly, it should be done in an environment that promotes psychological safety,^14^ as done in NASA.^15^

## Communication and psychological safety

Crew resource management (CRM) revolutionised how astronauts and ground teams communicate effectively with each other to solve potential minor or more serious issues. NASA actively encouraged that anyone could raise concerns about flight safety, regardless of their position or seniority in the team. The Apollo 13 astronauts’ ability to make an improvised life-saving carbon dioxide (CO_2_) filter using limited materials is a clear example of how this culture can promote excellent results in a highly stressful and potentially dangerous environment. Astronauts and engineers of varying positions were able to mitigate such a serious problem due to this safety-oriented culture.^15^

Operating theatres often have steep authority gradients. Using CRM guidelines such as having clear and defined roles, team briefings, and confident and assertive communication between team members can help create a culture similar to that of NASA.^16,17^

## Normalisation of deviance

The phrase “normalisation of deviance” was coined by Diane Vaughan, an American sociologist who used the Challenger Shuttle disaster as the first example of how adopting precarious practices or behaviours became normalised. This was before they had caused a clear and obvious failure.^18^ As previously discussed, engineers were aware of the O-ring erosion issue on Challenger but this was accepted due to a lack of previous immediate negative consequences. Tragically, seven crew members, including Christa McAuliffe – a school teacher – lost their lives when Challenger broke apart 73 seconds into its flight.

Surgical teams and wider healthcare can also succumb to normalisation of deviance.^19^ For example, not seeing patients preoperatively oneself, failure to actively engage with checklists or seeing these as a tick-box exercise, relying on poor verbal handovers and other issues may all become normalised, especially in high-pressure or time-dependent situations. These may seem like small deviations from standard practice with little or no immediate consequences but, as with Challenger, can lead to serious error and even fatality. Trolleys in the emergency department corridor is another example of how deviant practice has become ‘accepted’.

Surgical teams should ideally compare their work regularly with accepted systems to ensure they are practising at the highest possible standard. Checklists should become a more meaningful safety-orientated process rather than a tick-box exercise.^17^ NASA regularly reviews these nontechnical skills as well the technical performance of their staff. Can we say the same in surgery?

## Translating NASA practice into surgical practice

NASA’s aspiration to achieve a robust safety culture as outlined above can be applied directly to surgery. NASA also has ‘mandatory cross checking’, where two team members must confirm a decision aloud before proceeding with the intended action. Dual verification, for example, instrument and swab counting, does occur in the operating theatre, but this practice could also be beneficial during high-risk surgical procedures. Drug-related errors are one of the most common in healthcare.^2^ A randomised controlled trial found the detection rate for error detection was 46% higher when two separate healthcare workers double-checked medications, compared with error detection rates during single-staff member check procedures.^20^ This further reinforces the value of dual confirmation in high-risk settings.

Surgery has embraced simulation training in many areas but perhaps could learn from NASA models and concentrate more on the team rather than individual approach (and assessment). NASA uses simulation as an opportunity to test systems under failure scenarios. Before Apollo 11 (the first Moon Landing), crews rehearsed many problems, including communication failure or hardware faults. Surgical teams could adopt NASA’s approach, by including full rehearsals under realistic stress. These stressful situations could be due to sudden patient deterioration, major haemorrhage or other surgical complications. A study that implemented this style of simulation in laparoscopic surgery found there was a significant improvement in the team’s ability to recognise problems or errors that may be detrimental to patient safety, hence supporting the fact that this training can improve the team’s ability to function in the face of adversity.^21^

Every NASA mission is supported by ‘mission control’. This is a remote team who monitor live data and provide constant direction and advice to astronauts. Surgeons could adopt something similar by using digital infrastructure to provide remote senior support if required during operations. A systematic review of 66 studies on the impact of telementoring in surgery found it led to an increase in both surgeon competence and the quality of clinical outcomes in the operating theatre. The review also found that telementoring had a similar safety and efficacy profile to onsite mentoring.^22^

Finally, meticulous debriefs are routine practice in NASA. Independent of success, after every mission, all teams meet to analyse the mission from beginning to end. The primary focus is to improve systems rather than assign any potential blame. In surgery, debriefings are sometimes not done at all or are rushed at the end of the operating list. A systematic review found that surgical debriefs consistently improved team communication, psychological safety and clinical outcomes. It could be beneficial to embed regular, multidisciplinary debriefs after all operating lists.^23^

## Conclusion

NASA has experienced many failures and disasters with tragic loss of life. Their safety journey can be described as a progressive transformation from reactive, incident-driven responses to a proactive, learning-focused safety culture, shaped by both tragedy and innovation (Table 1). It reflects a long-standing commitment to protecting human life, mission success and continuous improvement in high-risk environments. NASA safety culture is now built upon a foundation of transparency, reporting, Just Culture, flexibility, learning and engagement, and a rigorous analysis of systems rather than individuals. By moving from individual blame and strengthening defence systems and promoting psychological safety, NASA has developed an environment that guarantees safety as its number one priority. This approach can help operating theatres become a safer for patients and a more enjoyable place to work for surgical teams and staff. To truly advance, surgery must move beyond reacting to failure and apportioning blame and become more proactive in designing systems to help prevent and learn from error.

**Table 1:** Chronology of NASA’s safety journey

Stage	Description
1. Early years: heroic risk, technology challenge (1958–1967)	• Safety not yet systematised; risk accepted as inherent to exploration • Emphasis on rapid progress
2. Institutionalising safety: learning from tragedy (1967–1986)	• Introduced formal safety protocols, checklists and hazard reviews • Apollo programme saw successful safety integration but became complacent, as Space Shuttle program began
3. Challenger disaster and cultural reckoning (1986)	• Seven astronauts died – ‘normalisation of deviance’ term introduced • Culture too hierarchical, with engineers’ concerns overridden by launch pressures
4. Rebuilding safety: safety and mission assurance (1986–2003)	• OSMA established • Improvements in risk assessment, reliability and communication introduced
5. Columbia disaster and the shift to a learning culture (2003)	• Shuttle foam risks known but not fully addressed • CAIB criticised ‘normalisation of deviance’ and organisational silence • Safety culture, leadership and decision-making re-evaluated
6. Evolving toward a Just and Learning Culture (2003+)	• Embraced Just Culture, HRO principles, and safety culture surveys • Focus expanded to include psychological safety, resilience and adaptive learning
7. Current state: proactive, integrated, and forward-looking (2025+)	• Safety is a core organisational value, embedded in both human spaceflight and robotic missions • Collaboration with commercial partners (e.g., SpaceX, Boeing) to co-develop safety standards • Investments in predictive analytics, digital twins, and AI for hazard detection and risk modelling • Emphasis on continuous learning, cross-agency knowledge sharing and mission assurance

AI = artificial intelligence; CAIB = Columbia Accident Investigation Board; HRO = High Reliability Organisation; OSMA = Office of Safety and Mission Assurance.
